# A two-phase comprehensive NSCLC prognostic study identifies lncRNAs with significant main effect and interaction

**DOI:** 10.1007/s00438-022-01869-3

**Published:** 2022-02-26

**Authors:** Jing Zhu, Jinxing Guan, Xinyu Ji, Yunjie Song, Xiaoshuang Xu, Qianqian Wang, Quanan Zhang, Renhua Guo, Rui Wang, Ruyang Zhang

**Affiliations:** 1 Department of Oncology, The Affiliated Jiangning Hospital of Nanjing Medical University, 169 Hushan Road, No. 2 Building, 212 East Ward, Nanjing, 211100 Jiangsu China; 2 Department of Biostatistics, Center for Global Health, School of Public Health, Nanjing Medical University, 101 Longmian Avenue, SPH Building, Room 406, Nanjing, 211166 Jiangsu China; 3 Department of Medical Oncology, The First Affiliated Hospital of Nanjing Medical University, 300 Guangzhou Road, No. 3 Building, Floor 10, Nanjing, 210003 Jiangsu China; 4 Department of Medical Oncology, Jinling Hospital, School of Medicine, Nanjing University, 34 Yanggongjing Street, Building 1, Floor 6, Nanjing, 210002 Jiangsu China

**Keywords:** lncRNA, Main effect, Interaction, Non-small-cell lung cancer, Overall survival

## Abstract

Long noncoding RNA (lncRNA) are involved in regulating physiological behaviors for various malignant tumors, including non-small-cell lung cancer (NSCLC). However, few studies comprehensively evaluated both lncRNA–lncRNA interaction effects and main effects of lncRNA on overall survival of NSCLC. Hence, we performed a two-phase designed study of lncRNA expression in tumor tissues using 604 NSCLC patients from The Cancer Genome Atlas as the discovery phase and 839 patients from Gene Expression Omnibus as the validation phase. In the discovery phase, we adopted a two-step strategy, *Screening before Testing*, for dimension reduction and signal detection. These candidate lncRNAs first screened out by the weighted random forest (Ranger), were then tested through the Cox proportional hazards model adjusted for covariates. Significant lncRNAs with either type of effects aforementioned were carried forward into the validation phase to confirm their significances again. As a result, in the discovery phase, 19 lncRNAs were identified by Ranger, among which five lncRNAs and one pair of lncRNA–lncRNA interaction exhibited significant effects (FDR-*q* ≤ 0.05) main and interaction effects on NSCLC survival, respectively, through Cox model. After the independent validation, we finally observed that one lncRNA (ENSG00000227403.1) with main effect was robustly associated with NSCLC prognosis (*HR*_discovery_ = 0.90, *P* = 1.20 × 10^–3^; *HR*_validation_ = 0.94, *P* = 4.11 × 10^–3^) and one pair of lncRNAs (ENSG00000267121.4 and ENSG00000272369.1) had significant interaction effect on NSCLC survival (*HR*_discovery_ = 1.12, *P* = 3.07 × 10^–4^; *HR*_validation_ = 1.11, *P* = 0.0397). Our comprehensive NSCLC prognostic study of lncRNA provided population-level evidence for further functional study.

**Supplementary Information:**

The online version contains supplementary material available at 10.1007/s00438-022-01869-3.

## Introduction

Lung cancer is the leading cause of cancer death worldwide (Sung et al. 2021). It is well known that majority of lung cancers are non-small cell lung cancer (NSCLC) in histological classification, including lung adenocarcinoma (LUAD) and lung squamous cell carcinoma (LUSC) (Travis 2020). In the half past century, great efforts have been made in the treatments of lung cancer, including surgery, chemotherapy, radiotherapy, targeted therapy, anti-angiogenic therapy, immunotherapy, etc. (Mok et al. 2009, 2019; Auperin et al. 2010). Therefore, the survival rate and quality of life of patients has been significantly improved (Wu et al. 2014; Mok et al. 2019; Sung et al. 2021). However, pathological mechanism of lung cancer progression still largely remains unclear (e.g. tumor cell invasion and drug resistance) (Herbst et al. 2018).

Long noncoding RNAs (lncRNAs) are a type of non-protein-coding transcript longer than 200 nucleotides and are one of the emerging regulators which are involved in diverse biological processes (Boon et al. 2016; Jandura and Krause 2017). Many studies have demonstrated that lncRNA plays an important role (e.g. oncogenes or tumor suppressors) in regulating the physiological behaviors of malignant tumors, including lung cancer (Schmitt and Chang 2016; Braicu et al. 2019; Xu et al. 2019). In recent years, accumulating evidences indicate that the interaction effects of lncRNAs are essential for the initiation and progression of cancers, such as lncRNA-protein interaction and lncRNA-miRNA-mRNA network (Ferrè et al. 2016; Wang et al. 2019). Especially, lncRNA-AC020978/PKM2/HIF-1α is a new perspective in the prevention or treatment of NSCLC (Hua et al. 2020). However, few studies ever focused on lncRNA–lncRNA interaction effects on NSCLC overall survival, which may provide pivotal clues for the biologic mechanisms of complex diseases (Zhang et al. 2019b) and enhance prediction accuracy (Chatterjee et al. 2016; Li et al. 2019).

Hence, we performed a comprehensive analysis of lncRNAs to evaluate their main effects and interaction effects on NSCLC survival through a two-phase designed study, using 604 NSCLC patients from The Cancer Genome Atlas (TCGA) as the discovery phase and 839 patients from Gene Expression Omnibus (GEO) as the validation phase.

## Materials and methods

### Data collection and study population

LncRNA expression data in tumor tissue, as well as demographic and clinical data, were retrieved from TCGA and GEO. (1) TCGA: The fragments per kilobase of per million (FPKM) value of lncRNA expression, as well as corresponding demographic and clinical information of 986 NSCLC patients, were downloaded from TCGA (https://portal.gdc.cancer.gov). (2) GEO: Totally, 6 available datasets consisting of 1096 NSCLC patients, profiled by the Affymetrix Human Genome U133A Plus 2.0 Array, were obtained from GEO (https://www.ncbi.nlm.nih.gov/geo/), which had the maximum overlapped lncRNAs to TCGA data, adequate sample sizes after quality control (e.g., *N* > 50) and, meanwhile raw data instead of standardized data, including GSE3141, GSE37745, GSE30219, GSE50081, GSE29013 and GSE31210.

LncRNA was annotated by gencode.v22 (https://www.gencodegenes.org/), and we obtained expression of 15,900 lncRNAs in TCGA. Before association analysis, we performed quality control procedures to acquire reliable lncRNA expression. Briefly, lncRNAs were excluded if they met any of the below criteria: all gene expression values equal to 0 or proportion of missing values is greater than 10%. Further, samples with missing values of any clinical variables were also excluded. Finally, 604 samples (294 LUAD and 310 LUSC) with 4313 lncRNAs in TCGA were retained in subsequent association analysis. In GEO, there were 839 patients (634 LUAD and 205 LUSC) remained after removing patients without complete clinical information. We performed a two-phase designed study of lncRNAs using subjects in TCGA as the discovery phase and subjects in GEO as the validation phase. The demographic and clinical information of subjects from two phases were described in Table 1.

**Table 1:** Demographic and clinical descriptions of NSCLC patients in the discovery and validation phases

Variable	Discovery phase (*N* = 604)	Validation phase (*N* = 839)
Age (years)	66.02 ± 9.55	63.51 ± 9.34
Gender, *N* (%)		
Male	355 (58.77%)	411 (56.38%)
Female	249 (41.23%)	318 (43.62%)
Unknown	0	110
Race, *N* (%)		
Asian	8 (1.32%)	0
Black	55 (9.11%)	0
White	541 (89.57%)	0
Unknown	0	839
Smoke status, *N* (%)		
Never	0	26 (6%)
Former	416 (68.87%)	243 (56.12%)
Current	188 (31.13%)	164 (37.88%)
Unknown	0	406
Smoking packs every year	47.38 ± 29.96	–
Clinical stage, *N* (%)		
I	328 (54.30%)	496 (68.04%)
II	171 (28.31%)	172 (23.59%)
III	88 (14.57%)	53 (7.27%)
IV	17 (2.81%)	8 (1.10%)
Unknown	0	110
Histology, *N* (%)		
LUAD	294 (48.68%)	634 (75.57%)
LUSC	310 (51.32%)	205 (24.43%)
Survival year		
Median (95% CI)	3.91 (3.33–4.93)	7.53 (6.30–8.92)
Censoring rate	59.60%	55.66%

The validation phase consists of six datasets from GEO, including GSE3141 (*N* = 110), GSE37745 (*N* = 172), GSE30219 (*N* = 104), GSE50081 (*N* = 172), GSE31210 (*N* = 226) and GSE29013 (*N* = 55)

### Statistical analysis

**Fig. 1:**
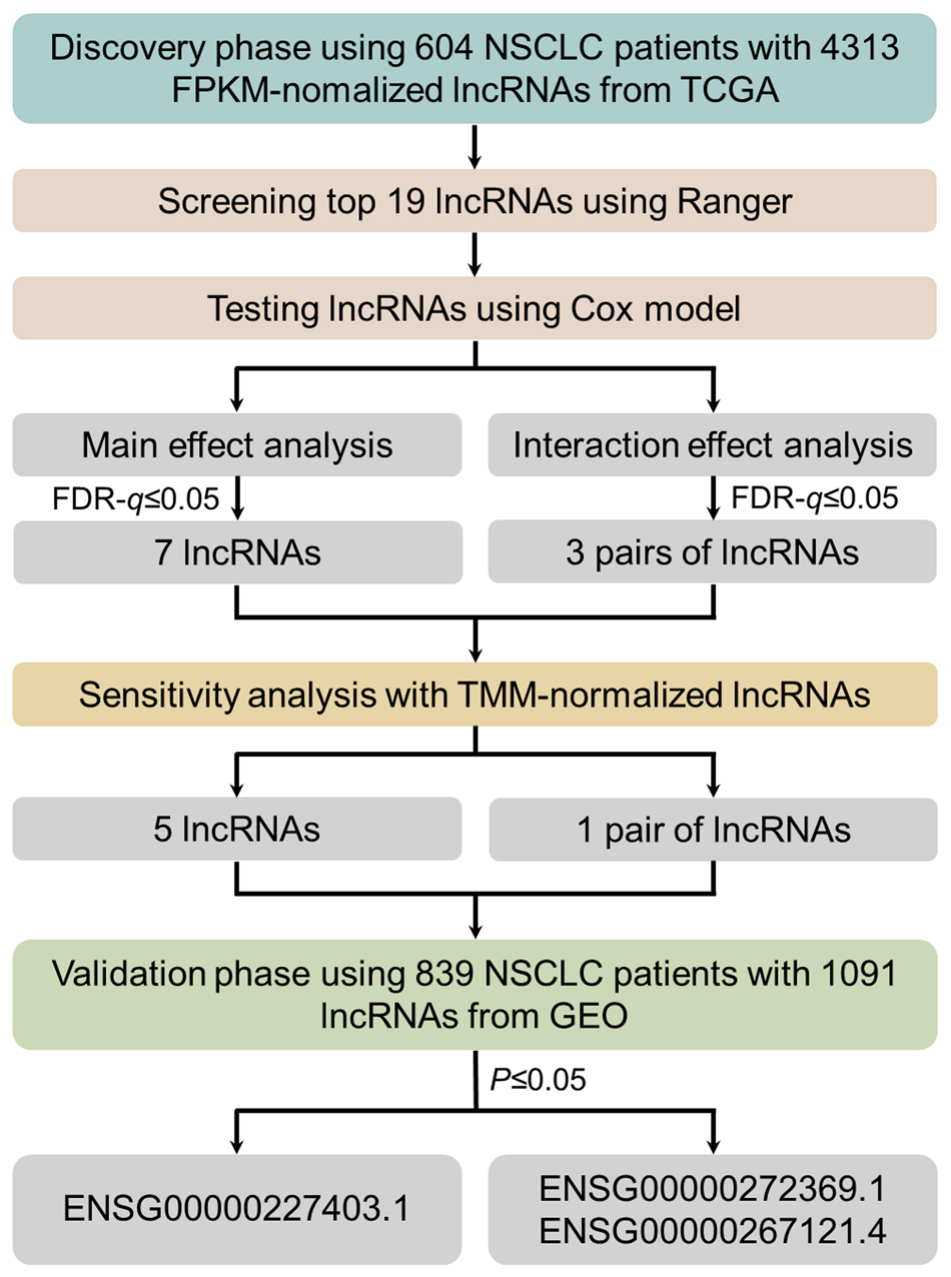
Statistical analysis work flow

The entire statistical analysis workflow was given in Fig. 1.

#### Screening before Testing strategy in the discovery phase

In the discovery phase, we adopted a two-step strategy, named *Screening before Testing*, for dimension reduction and signal detection. In the screening step, Ranger, a weighted version of random forest for analyzing time-to-event data, while adjusted for covariates, was employed to evaluate the importance of each individual lncRNA (Breiman 2001), using R package *ranger*. A weight of 100% was assigned to each covariate to ensure all covariates were adjusted in each classification tree, including age, gender, race, clinical stage, smoking status and pack-year of smoking. Variable importance score (VIS) of each lncRNA was estimated and ranked in a descending order. The sliding windows sequential forward feature selection (SWSFS) algorithm was then applied to identify the top important lncRNAs (Jiang et al. 2009). The SWSFS algorithm incorporated these lncRNAs one by one into the Ranger model by the order of VIS. Then, we plotted the out of bagging (OOB) error rate, which measured the performance of each model consisting of top *k* lncRNAs. The top candidate lncRNAs were screened out for further analysis when the Ranger model reaching the lowest OOB error rate.

In the testing step, we further evaluated both main effects and lncRNA–lncRNA interaction effects of these top candidate lncRNAs, through Cox proportional hazards model adjusted for the same covariates aforementioned, using the R package *survival*. For main effect analysis and interaction effect analysis, we adopted the model 1 and 2 below, respectively.

$$\begin{gathered} h(t) = h_{0} (t)\exp \left( {\alpha_{1} \times {\text{lncRNA}} + \sum {\beta_{i} \times {\text{Covariate}}_{i} } } \right) \hfill \\ h(t) = h_{0} (t)\exp \left( {\alpha_{1} \times {\text{lncRNA}}_{1} { + }\alpha_{2} \times {\text{lncRNA}}_{2} { + }\alpha_{3} \times {\text{lncRNA}}_{1} \times {\text{lncRNA}}_{2} { + }\sum {\beta_{i} \times {\text{Covariate}}_{{\text{i}}} } } \right) \hfill \\ \end{gathered}$$

The association results were described as hazard ratio (HR) and 95% confidence interval (CI). Multiple comparisons were adjusted using false discovery rate method (FDR; measured by FDR-*q* value) (Klipper-Aurbach et al. 1995). LncRNAs with significant (FDR-*q* ≤ 0.05) main effects or interaction effects on NSCLC survival were reserved for subsequent analysis.

To retain the robustly significant association between lncRNA and NSCLC survival, we additionally downloaded raw counts of lncRNA expression from TCGA and calculated a trimmed mean of *M*-values (TMM) between each pair of samples to adjust the library sizes (Robinson and Oshlack 2010), by R package *DGEobj.utils*. Then, we performed sensitivity analysis of TMM-normalized expression data for top candidate lncRNAs and significant (FDR-*q* ≤ 0.05) lncRNAs with either type of effects aforementioned were preserved. Finally, only these lncRNAs simultaneously significant in both FPKM and TMM data were carried forward into the validation phase to confirm their significances once again.

#### Trans-platform pseudo-validation strategy in the validation phase

Due to the difference of gene expression platforms between TCGA and GEO, some significant lncRNAs identified in TCGA were not profiled in GEO. Therefore, we performed trans-platform pseudo-validation using their surrogate lncRNAs, which had significant (FDR-*q* ≤ 0.05) and maximum correlation with the targets. The main effects and interaction effects of these surrogate lncRNAs were again tested by Cox proportional hazards model adjusted for covariates. Finally, only these lncRNAs were preserved if they met both criteria below: (1) *P* ≤ 0.05 in the validation phase, and (2) consistent effects across both discovery and validation phases.

Furthermore, the significances of these lncRNAs were evaluated in LUAD and LUSC subgroup populations. Besides, we compared two models to highlight the contribution of lncRNAs to the prognostic prediction of NSCLC survival: (1) a basic model with merely demographic and clinical information, and (2) an optimized model added significant lncRNAs with either main effects or interaction effects. We predicted 3- and 5-year overall survival of NSCLC patients using the Kaplan–Meier method for time-to-event data (Heagerty et al. 2000). The accuracy of the prediction was presented using a receiver operating characteristic (ROC) curve and was measured by time-dependent area under the ROC curve (AUC) by the R package *survivalROC*. The 95% CI and *P* value of the AUC improvement were calculated on the basis of 1,000-time bootstrap resampling.

Continuous variables were summarized as mean ± standard deviation (SD), and categorized variables were described by frequency (*n*) and proportion (%). Statistical analyses were performed using R version 3.6.3 (The R Foundation of Statistical Computing, Vienna, Austria).

## Result

### The two-phase study identified one lncRNA with significant main effect and one pair of lncRNAs with significant interactions

In the discovery phase, we identified top 19 lncRNAs which together had the lowest OOB error in the screening step by the SWSFS algorithm (Figure S1, S2). In the testing step, we observed five lncRNAs (ENSG00000227403.1, ENSG00000273038.2, ENSG00000269609.4, ENSG00000273230.1 and ENSG00000204949.7) and one pair of lncRNAs (ENSG00000272369.1 and ENSG00000267121.4) had robust and significant (FDR-*q* ≤ 0.05) main effects and interaction effects on NSCLC survival, respectively (Table S1). The annotation information was presented in Table S2. Compared to the basic model, the optimized model added these lncRNAs with either type of effects had significant improved prediction accuracy (3-year survival: AUC_optimized_ = 0.72 *vs* AUC_basic_ = 0.65, 12.0% increase, *P* < 2.2 × 10^–16^; 5-year survival: AUC_optimized_ = 0.74 vs AUC_basic_ = 0.63, 16.9% increase, *P* < 2.2 × 10^–16^) (Figure S3).

In the validation phase (Table S3), we finally observed one lncRNA, ENSG00000227403.1, significantly associated with NSCLC survival (*HR*_discovery_ = 0.90, *P* = 1.20 × 10^–3^; *HR*_validation_ = 0.94, *P* = 4.11 × 10^–3^) and one pair of lncRNAs (ENSG00000267121.4 and ENSG00000272369.1) had significant interaction effect on NSCLC survival (*HR*_discovery_ = 1.12, P = 3.07 × 10^–4^; *HR*_validation_ = 1.11, *P* = 0.0397) (Table 2).

**Table 2:** Association results of one lncRNA and one pair of lncRNAs derived from Cox proportional hazards model adjusted for covariates in main effect and interaction effect analyses

Type of analysis	LncRNA	Discovery phase		Validation phase	
		HR (95% CI)	*P*	HR (95% CI)	*P*
Main effect	ENSG00000227403.1	0.90 (0.84, 0.96)	1.20 × 10^–3^	0.94 (0.90, 0.98)	4.11 × 10^–3^
Interaction effect	ENSG00000272369.1	0.69 (0.57, 0.83)	8.28 × 10^–5^	0.44 (0.24, 0.79)	6.41 × 10^–3^
	ENSG00000267121.4	1.30 (1.12, 1.51)	5.61 × 10^–4^	0.38 (0.17, 0.88)	0.0228
	Interaction term	1.12 (1.05, 1.19)	3.07 × 10^–4^	1.11 (1.01, 1.23)	0.0397

In the validation phase, the surrogates of ENSG00000227403.1, ENSG00000272369.1 and ENSG00000267121.4 were ENSG00000253738.1, ENSG00000265666.1 and ENSG00000227039.5, respectively

### The lncRNA with significant main effect substantially discriminated subjects at high risk of mortality from NSCLC patients

**Fig. 2:**
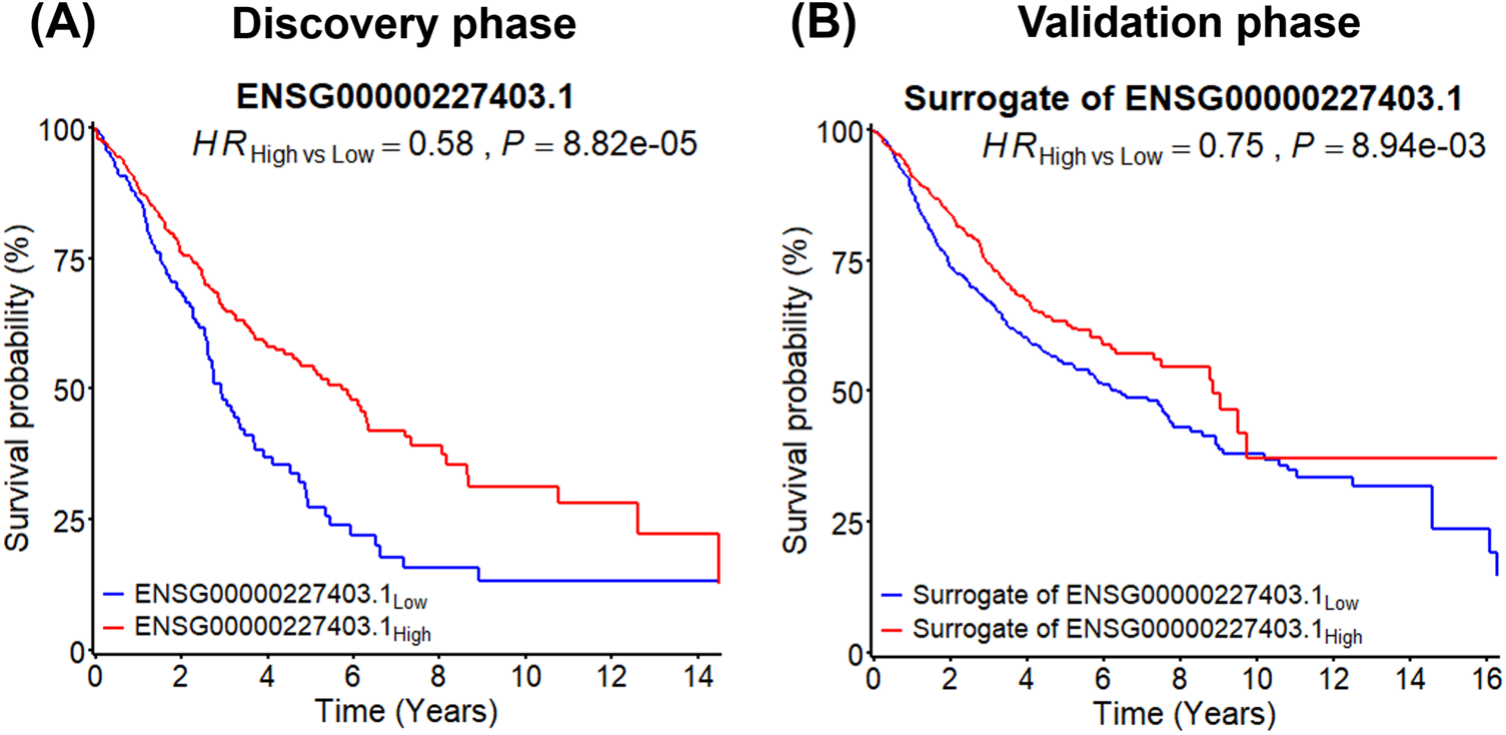
Kaplan–Meier survival curves for patients with low and high expression of ENSG00000227403.1 in the discovery and validation phases

NSCLC patients were divided into low and high expression groups based on median value of ENSG00000227403.1. By comparison of the Kaplan–Meier survival curves between two groups (Fig. 2), these patients in high expression group had significant better overall survival in the discovery phase (*HR*_high vs low_ = 0.58, *P* = 8.82 × 10^–5^) and the validation phase (*HR*_high vs low_ = 0.75, *P* = 8.94 × 10^–3^), indicating that subjects with low expression of ENSG00000227403.1 were at high risk of mortality.

### The effect of one lncRNA on NSCLC survival was modified by another lncRNA

**Fig. 3:**
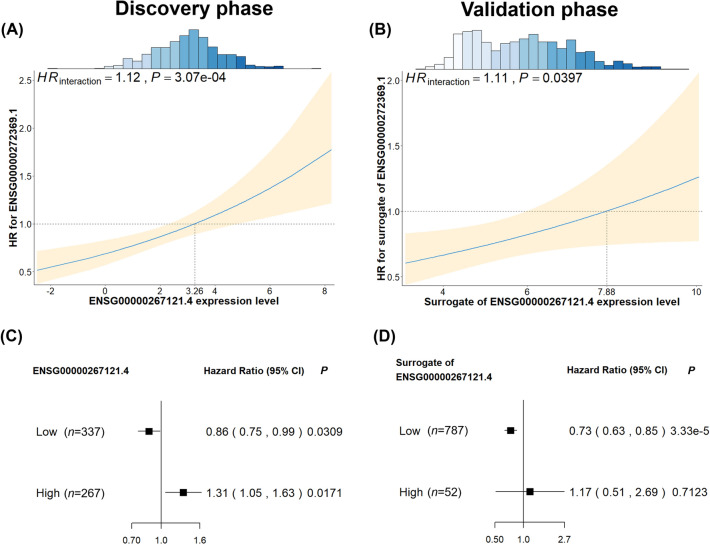
Line and forest plots illustrating lncRNA–lncRNA interaction effect between ENSG00000267121.4 and ENSG0000027236.9 on NSCLC survival

For the interaction between ENSG00000272369.1 and ENSG00000267121.4, we observed that, with increased expression level of ENSG00000267121.4, there was an elevated effect of ENSG00000272369.1 on NSCLC survival in the discovery phase (Fig. 3A) and the validation phase (Fig. 3B). Therefore, ENSG00000267121.4 was a modifier of the association between ENSG00000272369.1 and NSCLC survival. Besides, to better understand this interaction, patients were divided into low and high expression groups based on their ENSG00000267121.4 values, using cutoff value 3.26 and 7.88 in the discovery and validation phase, respectively. We observed varied effects of ENSG00000272369.1 between different ENSG00000267121.4 expression groups. High expression of ENSG00000272369.1 exhibited a significantly protective effect on NSCLC for these patients in low expression group of ENSG00000267121.4 in the discovery phase (*HR* = 0.86, *P* = 0.0309) and the validation phase (*HR* = 0.73, *P* = 3.33 × 10^–5^) (Fig. 3C, D). However, the effect of ENSG00000272369.1 was reversed for these patients in high expression group of ENSG00000267121.4 in the discovery phase (*HR* = 1.31, *P* = 0.0171) and the validation phase (*HR* = 1.17, *P* = 0.7123).

**Fig. 4:**
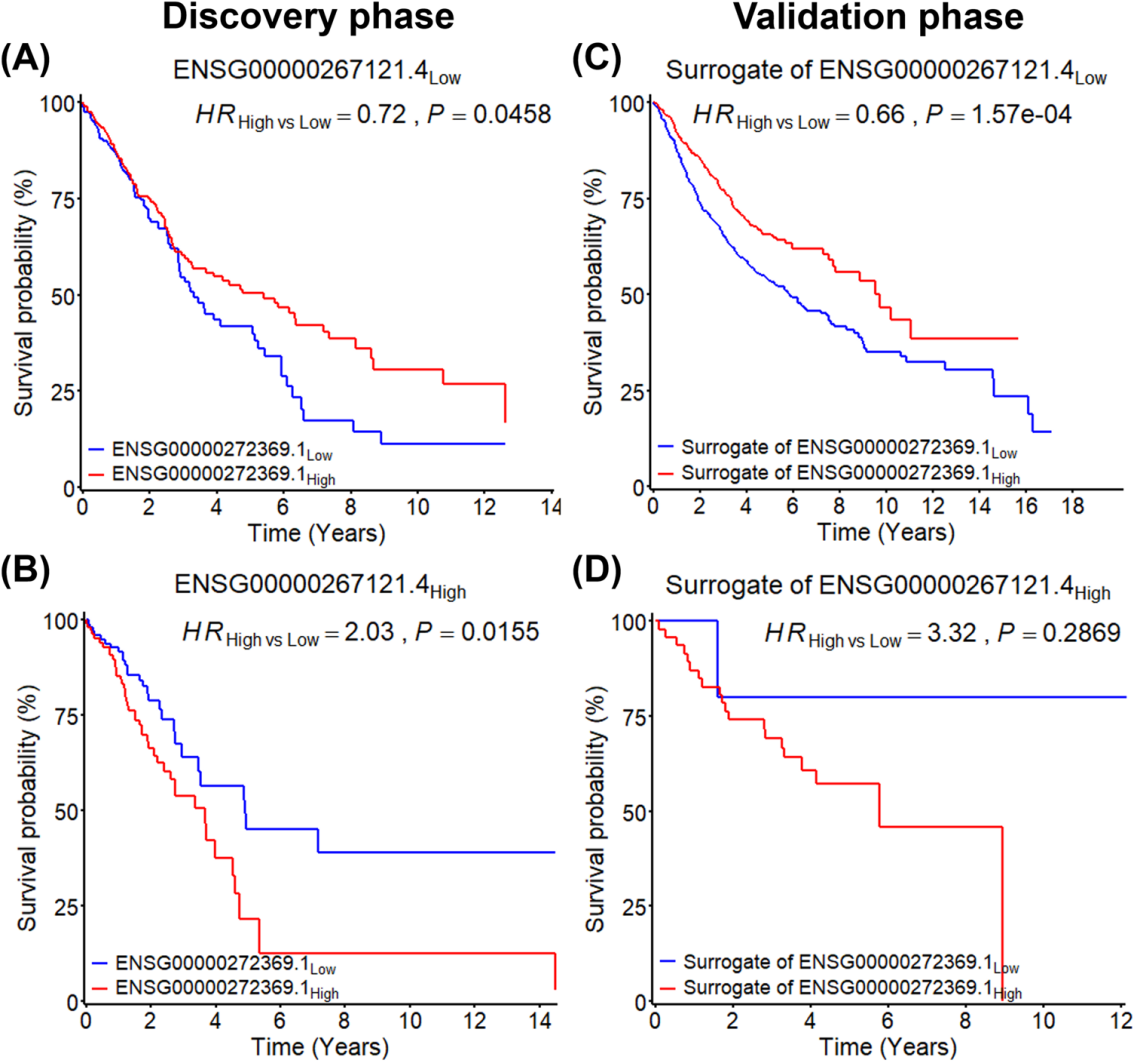
Kaplan–Meier survival curves for patients having different expression levels of two lncRNAs

To visualize the heterogeneous effect of ENSG00000272369.1 between two expression groups of ENSG00000267121.4, we further categorized ENSG00000272369.1 into dichotomous variable by its median value. In low expression group of ENSG00000267121.4, subjects with high expression of ENSG00000272369.1 had significantly better survival compared to these with low ENSG00000272369.1 in the discovery phase (*HR*_high vs low_ = 0.72, *P* = 0.0458) (Fig. 4A) and the validation phase (*HR*_high vs low_ = 0.66, *P* = 1.57 × 10^–4^) (Fig. 4C). On the contrary, in high expression group of ENSG00000267121.4, subjects with high expression of ENSG00000272369.1 had worse survival in two phases (Fig. 4B, D).

The stratified analysis by histology confirmed the significance of main effect of ENSG00000227403.1 on NSCLC survival (Table S4), except for LUSC subgroup with small sample size in the validation phase. Besides, the interaction effect between ENSG00000272369.1 and ENSG00000267121.4 maintained significance only in LUAD subgroup in the discovery phase.

## Discussion

Non-coding RNAs (ncRNAs), accounting for 98% of the human genome, are classified into several types including: lncRNAs, micro RNAs (miRNAs), circular RNA (circRNAs) and so on (Zhang et al. 2020a; b). Human lncRNAs are abundant and diverse, and nearly 53,000 different lncRNAs are known but only about 1000 are present in sufficiently high copy number to authentically justify their functional importance (Djebali et al. 2012). In recent years, extensive evidence has suggested that lncRNAs are involved in the occurrence of many diseases, including cancer (Liang et al. 2018). LncRNAs have been shown to participate in the development, progression, proliferation, and invasion of NSCLC used variety of ways (Zhang et al. 2019c; Chen et al. 2020a, b).

Emerging evidence indicates that there exist many types of interactions associated with NSCLC survival, including gene–gene (Zhang et al. 2019b), gene-smoking (Zhang et al. 2019a), gene-age (Chen et al. 2020a; b) and gene-histology interactions (Ji et al. 2020). As is well known, interactions provide important clues for the biologic mechanisms and heritability of complex diseases (Trerotola et al. 2015). Thus, biomarkers with main effects only explained a small proportion of the phenotypic variations and the GxG interactions may be one of the important reasons accounting for the missing heritability (Trerotola et al. 2015). The studies of lncRNAs account for 13% of the total studies of ncRNAs in lung cancer until year 2019 (Braicu et al. 2019). Anyway, a mass of studies only focused on the association between lncRNAs and lung cancer, by testing their main effects. GxG interaction analysis provides a different way of identifying new biomarkers (Cordell 2009), which can further improve the predictive accuracy of statistical models (Cordell 2009; Zhang et al. 2020a, b), or offer statistical evidence for functional studies. To our knowledge, this perhaps was the first attempt to explore the association between lncRNA–lncRNA interaction and NSCLC survival in population level. But functional experiments are still warranted to elaborate underlying mechanism.

For the three lncRNAs (ENSG00000227403.1, ENSG00000267121.4 and ENSG00000272369.1) successfully validated in an independent population, previous study indicated that *AC009299.3*, to which ENSG00000227403.1 mapped, was involved in the control of autophagy (Zhu et al. 2018; Wu et al. 2021). Meanwhile, high expression of this gene was also associated with better LUAD prognosis (Wu et al. 2021). Besides, *CTD-2020K17.1*, where ENSG00000267121.4 located, has been identified to promote migration, invasion, and proliferation of ovarian cancer (Zhu et al. 2018). Although these three lncRNAs lack explicit functional level evidence relevant to NSCLC survival, we provided robust and significant population-level evidence for further mechanistic study.

For other four lncRNAs (ENSG00000204949.7, ENSG00000273038.2, ENSG00000269609.4 and ENSG00000273230.1) failed in the independent validation, *FAM83A-AS1* (ENSG00000204949.7) and *RP11-479G22.8* (ENSG00000273038.2) play essential roles in the development and progression of several cancers, including lung cancer, esophageal cancer and hepatocellular carcinoma (Wei and Zhang 2016; Wu et al. 2018; He and Yu 2019; Shi et al. 2019; Huang et al. 2020; Jia et al. 2021). Besides, *RPARP-AS1* (ENSG00000269609.4) could promote the proliferation, migration and invasion of tumor cells through sponging mir-125a-5p, which had been proved functional in the growth, invasion and metastasis of lung cancer and other cancers (Naidu et al. 2017; Ren et al. 2021).

Our study has several strengths. First, our study simultaneously evaluated both main effects of lncRNAs and lncRNA–lncRNA interaction effects on NSCLC survival, which was a comprehensive prognostic study of lncRNAs. And, to our knowledge, this is perhaps the first lncRNA–lncRNA interaction study of NSCLC survival. Besides one lncRNA with significant main effect, we additionally identified one pair of lncRNAs which exhibited significant interaction effect, providing potential evidence that complex disease (e.g., lung cancer) was driven by complex association pattern. Second, we utilized a two-phase study design to control the false positives, where statistical significance was corrected using FDR method in the discovery phase and again confirmed in the validation phase. Third, we adopted a two-step strategy, Screening before Testing, to improve the calculation speed of analysis and boost the statistical power of analysis in high dimensional scenario. Meanwhile, covariates were adjusted in both screening and testing steps to obtain more roust association results.

We also acknowledge some limitations. First, the significant lncRNAs identified in TCGA happen to be not profiled in GEO. Thus, we compromised by a trans-platform pseudo-validation using surrogate lncRNAs of these target lncRNAs. Even though one lncRNA and one pair of lncRNAs were successfully validated, additional available public database and further studies of target lncRNAs are still warranted. Second, the censoring rate of time-to-event data is high in TCGA and GEO, which may result in low statistical power in analysis. Thus, we only focused on two-way interaction between pair of lncRNAs. Third, further functional experiments of lncRNAs are warranted to provide biological evidence, beyond our statistical evidence. Thus, the association still should be interpreted with caution.

## Conclusion

Our two-phase comprehensive NSCLC prognostic study of lncRNAs identified one lncRNA (ENSG00000227403.1) with significant main effects and one pair of lncRNAs (ENSG00000267121.4 and ENSG00000272369.1) with significant interaction effects on overall survival, providing population-level evidence for further functional study.

## Supplementary Information

Below is the link to the electronic supplementary material.

Supplementary file1 (DOCX 293 KB)

## Data Availability

The datasets used in the current study are available from the corresponding author on reasonable request.

## Code availability

The datasets used in the current study are available from the corresponding author on reasonable request.
