# Supplementary material for: A two-phase comprehensive NSCLC prognostic study identifies lncRNAs with significant main effect and interaction
Source: Mol Genet Genomics. 2022 Feb 26;297(2):591–600. doi: 10.1007/s00438-022-01869-3 (PMC8960609; doi:10.1007/s00438-022-01869-3)

**Table S1.** Association results for seven lncRNAs and three pair of lncRNAs derived from Cox proportional hazards model adjusted for covariates in main effect and interaction analyses of top 19 lncRNAs identified by the SWSFS algorithm in the discovery phase.

| Type of analysis | LncRNA | Results from FPKM-normalized lncRNA data | | | | Results from TMM-normalized lncRNA data | | | |
| --- | --- | --- | --- | --- | --- | --- | --- | --- | --- |
|  |  | HR (95% CI) | *z* | *P* | FDR-*q* | HR (95% CI) | *z* | *P* | FDR-*q* |
| Main effect | ENSG00000227403.1 | 0.73 (0.62, 0.86) | -3.708 | 2.09×10^-4^ | 8.68×10^-4^ | 0.90 (0.84, 0.96) | -3.233 | 1.23×10^-3^ | 4.66×10^-3^ |
|  | ENSG00000273038.2 | 1.55 (1.23, 1.95) | 3.728 | 1.93×10^-4^ | 8.68×10^-4^ | 1.27 (1.11, 1.44) | 3.623 | 2.91×10^-4^ | 2.00×10^-3^ |
|  | ENSG00000269609.4 | 0.67 (0.51, 0.89) | -2.776 | 5.50×10^-3^ | 0.0149 | 0.69 (0.56, 0.84) | -3.717 | 2.02×10^-4^ | 2.00×10^-3^ |
|  | ENSG00000273230.1 | 1.52 (1.23, 1.89) | 3.825 | 1.31×10^-4^ | 8.68×10^-4^ | 1.24 (1.10, 1.39) | 3.468 | 5.24×10^-4^ | 2.49×10^-3^ |
|  | ENSG00000204949.7 | 1.23 (1.10, 1.38) | 3.685 | 2.28×10^-4^ | 8.68×10^-4^ | 1.07 (1.01, 1.13) | 2.360 | 0.0183 | 0.0482 |
|  | ENSG00000258919.1 | 1.44 (1.18, 1.75) | 3.606 | 3.11×10^-4^ | 9.83×10^-4^ | 1.07 (1.00, 1.15) | 2.068 | 0.0386 | 0.0733 |
|  | ENSG00000279841.1 | 1.58 (1.25, 2.00) | 3.830 | 1.28×10^-4^ | 8.68×10^-4^ | 1.04 (0.94, 1.15) | 0.815 | 0.4153 | 0.4384 |
| Interaction effect | ENSG00000272369.1 | 0.64 (0.42, 0.97) | -2.082 | 0.0374 |  | 0.69 (0.57, 0.83) | -3.936 | 8.28×10^-5^ |  |
|  | ENSG00000267121.4 | 0.76 (0.56, 1.05) | -1.670 | 0.0949 |  | 1.30 (1.12, 1.51) | 3.450 | 5.61×10^-4^ |  |
|  | Interaction term | 1.50 (1.25, 1.80) | 4.297 | 1.73×10^-5^ | 0.0030 | 1.12 (1.05, 1.19) | 3.610 | 3.07×10^-4^ | 0.0175 |
|  | ENSG00000273230.1 | 1.13 (0.86, 1.47) | 0.874 | 0.3823 |  | 1.27 (1.08, 1.50) | 2.855 | 4.30×10^-3^ |  |
|  | ENSG00000232907.6 | 0.21 (0.08, 0.59) | -2.983 | 0.0028 |  | 0.59 (0.26, 1.36) | -1.235 | 0.2168 |  |
|  | Interaction term | 2.69 (1.79, 4.03) | 4.776 | 1.79×10^-6^ | 0.0003 | 1.07 (0.94, 1.22) | 0.984 | 0.3253 | 0.7947 |
|  | ENSG00000231290.4 | 0.94 (0.68, 1.30) | -0.388 | 0.6983 |  | 0.97 (0.86, 1.10) | -0.417 | 0.6764 |  |
|  | ENSG00000204949.7 | 1.13 (1.00, 1.28) | 1.922 | 0.0545 |  | 1.09 (1.03, 1.16) | 2.826 | 4.71×10^-3^ |  |
|  | Interaction term | 1.38 (1.18, 1.62) | 3.931 | 8.47×10^-5^ | 0.0145 | 1.02 (1.00, 1.04) | 2.023 | 0.0430 | 0.3680 |

**Table S2.** The annotation information for five lncRNAs with significant main effects and one pair of lncRNAs with significant interaction effects.

| LncRNA name | Location | Gene symbol | Biotype |
| --- | --- | --- | --- |
| ENSG00000273038.2 | chr10:32887255-32889311 | *RP11-479G22.8* | lincRNA |
| ENSG00000269609.4 | chr10:102449817-102461106 | *RPARP-AS1* | processed_transcript |
| ENSG00000273230.1 | chr7:1464497-1467522 | *RP11-1246C19.1* | lincRNA |
| ENSG00000204949.7 | chr8:123201172-123202743 | *FAM83A-AS1* | antisense |
| ENSG00000227403.1 | chr2:161244739-161249050 | *AC009299.3* | lincRNA |
| ENSG00000272369.1 | chr12:46537502-46652550 | *RP11-446N19.1* | lincRNA |
| ENSG00000267121.4 | chr17:45190931-45222222 | *CTD-2020K17.1* | antisense |

**Table S3.** Association results for five lncRNAs and one pair of lncRNAs derived from Cox proportional hazards model adjusted for covariates in main effect and interaction analyses in the validation phase.

| Type of analysis | Target lncRNA | Surrogate lncRNA | Surrogate gene symbol | *HR* (95% CI) | *z* | *P* |
| --- | --- | --- | --- | --- | --- | --- |
| Main effect | ENSG00000227403.1 | ENSG00000253738.1 | *OTUD6B-AS1* | 0.94 (0.90, 0.98) | -2.869 | 4.11×10^-3^ |
|  | ENSG00000273038.2 | ENSG00000260804.3 | *PKI55* | 1.00 (0.91, 1.11) | 0.082 | 0.9349 |
|  | ENSG00000269609.4 | ENSG00000269609.4 | *RPARP-AS1* | 1.11 (1.03, 1.20) | 2.704 | 6.85×10^-3^ |
|  | ENSG00000273230.1 | ENSG00000258634.3 | *RP4-773N10.4* | 0.87 (0.81, 0.94) | -3.624 | 2.91×10^-4^ |
|  | ENSG00000204949.7 | ENSG00000237523.1 | *LINC00857* | 1.09 (0.99, 1.20) | 1.698 | 0.0894 |
| Interaction effect | ENSG00000272369.1 | ENSG00000265666.1 | *RARA-AS1* | 0.44 (0.24, 0.79) | -2.726 | 6.41×10^-3^ |
|  | ENSG00000267121.4 | ENSG00000227039.5 | *ITGB2-AS1* | 0.38 (0.17, 0.88) | -2.277 | 0.0228 |
|  | Interaction term |  |  | 1.11 (1.01, 1.23) | 2.057 | 0.0397 |

**Table S4.** Association results of one lncRNA and one pair of lncRNAs derived from Cox proportional hazards model adjusted for covariates in main effect and interaction effect analyses stratified by histology.

| Type of analysis | Histology | LncRNA | Discovery phase | | Validation phase | |
| --- | --- | --- | --- | --- | --- | --- |
|  |  |  | HR (95 % CI) | *P* | HR (95 % CI) | *P* |
| Main effect | LUAD | ENSG00000227403.1 | 0.85 (0.76, 0.96) | 6.04×10^-4^ | 0.90 (0.86, 0.93) | 2.47×10^-7^ |
|  | LUSC | ENSG00000227403.1 | 0.90 (0.84, 0.97) | 6.49×10^-3^ | 1.04 (0.97, 1.16) | 0.2645 |
| Interaction effect | LUAD | ENSG00000272369.1 | 0.62 (0.54, 0.72) | 5.76×10^-10^ | 0.93 (0.83, 1.03) | 0.1614 |
|  |  | ENSG00000267121.4 | 1.26 (1.06, 1.49) | 9.60×10^-3^ | 1.19 (0.89, 1.60) | 0.2424 |
|  |  | Interaction term | 1.17 (1.09, 1.25) | 7.06×10^-6^ | 0.98 (0.94, 1.01) | 0.1798 |
|  | LUSC | ENSG00000272369.1 | 0.78 (0.50, 1.23) | 0.2846 | 0.98 (0.84, 1.13) | 0.7390 |
|  |  | ENSG00000267121.4 | 1.32 (0.95, 1.81) | 0.0948 | 0.89 (0.62, 1.30) | 0.5527 |
|  |  | Interaction term | 1.07 (0.92, 1.25) | 0.3559 | 1.01 (0.98, 1.06) | 0.4645 |

In the validation phase, the surrogates of ENSG00000227403.1, ENSG00000272369.1 and ENSG00000267121.4 were ENSG00000253738.1, ENSG00000265666.1 and ENSG00000227039.5, respectively.

**Figure S1.** Out of bag (OOB) error rate of top *k* lncRNAs in Ranger model through the sliding windows sequential forward feature selection (SWSFS) algorithm.


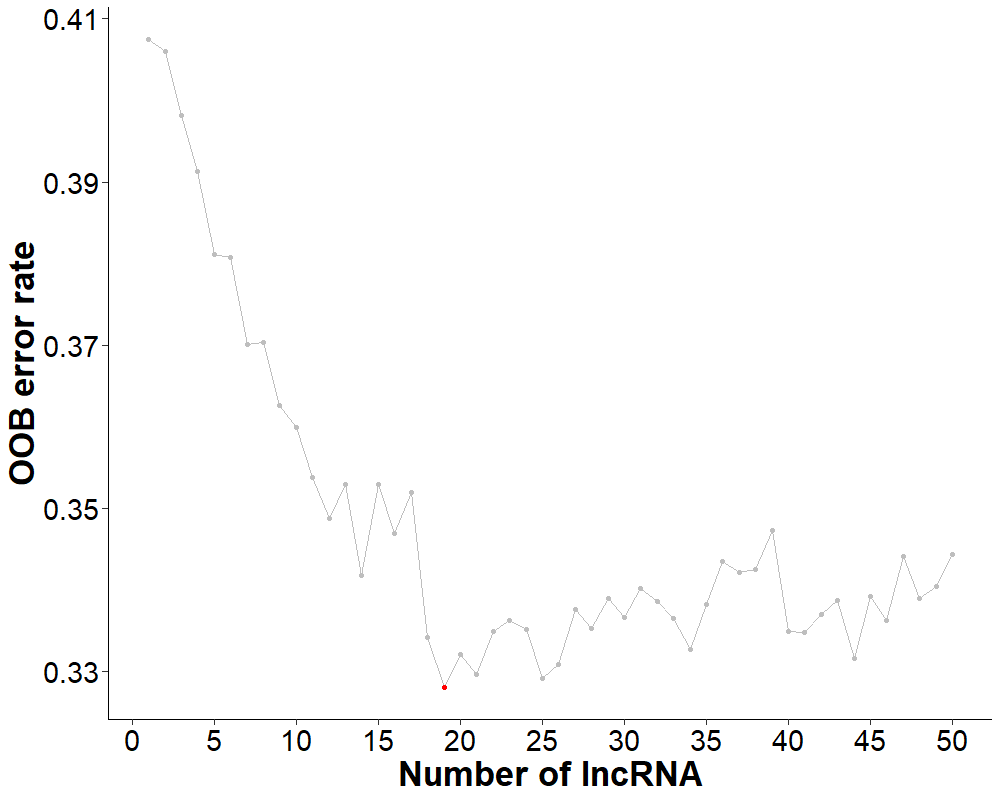


**Figure S2.** Top 19 lncRNAs ranked by variable importance score (VIS).


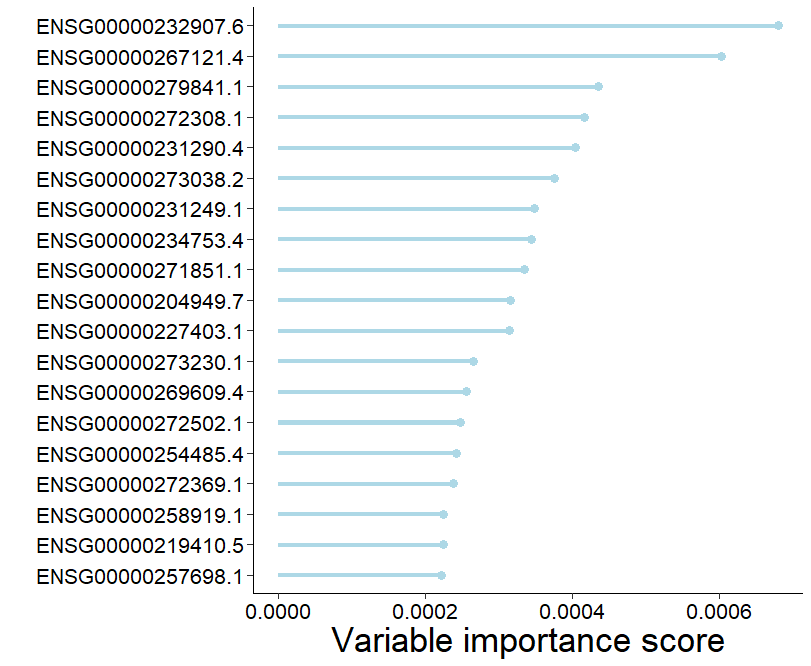


**Figure S3.** Time-dependent receiver operating characteristic curve (ROC) for 3- and 5-year survival prediction models.


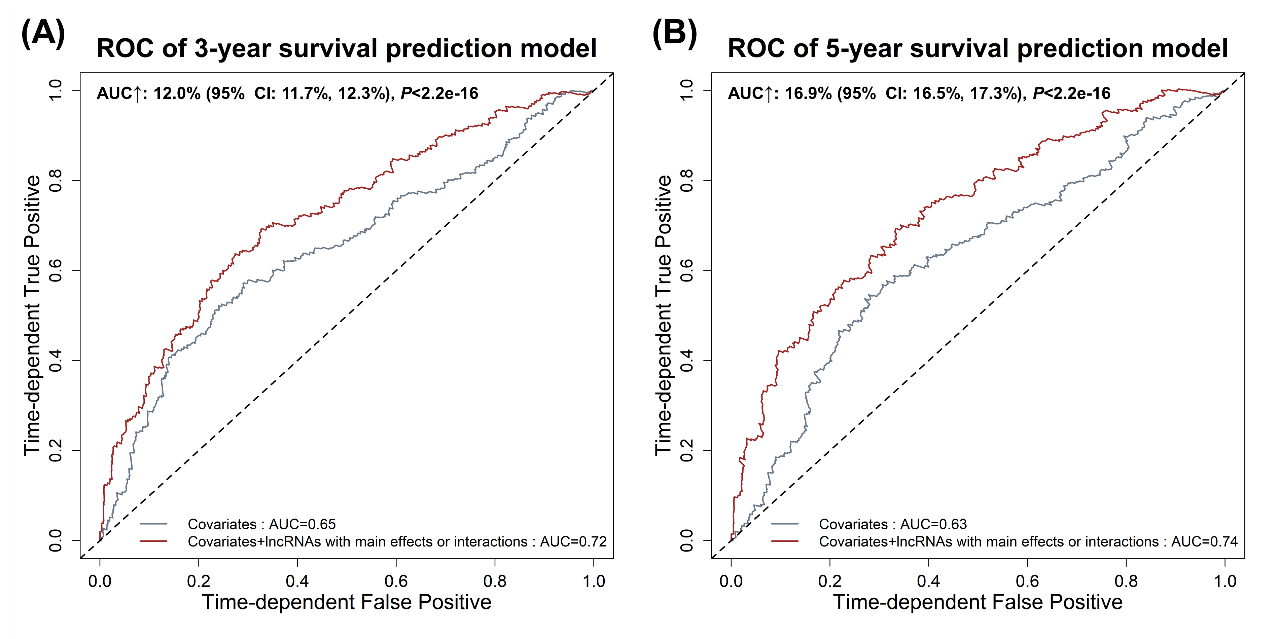

Supplement: Supplementary file 1 — Supplementary file1 (DOCX 293 KB) [file 438_2022_1869_MOESM1_ESM.docx]
